# Interleukin-6 as a therapeutic target in locomotor disorders

**DOI:** 10.1186/ar3592

**Published:** 2012-02-09

**Authors:** Norihiro Nishimoto, Miho Murakami, Takaji Matsutani, Jun Hashimoto, Nobuhiro Takagi

**Affiliations:** 1 Laboratory of Immune Regulation, Wakayama Medical University, Ibaraki, 567-0085, Japan; 2 Department of Orthopaedic Surgery, NHO Osaka-Minami Medical Center, Kawachinagano, 586-8521, Japan; 3 Chugai Pharmaceutical Co Ltd, Tokyo, Japan

## 

Interleukin-6 (IL-6) is a multifunctional cytokine that regulates immune response, inflammation, and hematopoiesis. Although IL-6 plays several important physiological roles, deregulated overproduction of IL-6 causes various clinical symptoms and laboratory abnormalities. In the locomotor disorders such as rheumatoid arthritis (RA) and juvenile idiopathic arthritis (JIA), IL-6 overproduction has been shown to be involved in inflammatory manifestations as well as joint destruction. Thus the blocking IL-6 signaling may be a therapeutic approach in those diseases. Various therapeutic antibodies targeting IL-6 have been developed, and tocilizumab (TCZ), an anti-IL-6 receptor antibody, precedes the others in clinical use.

TCZ, even in monotherapy, has been demonstrated to induce DAS28 remission frequently in patients with RA and suppress the radiographic progression of joint damage. TCZ more significantly reduced radiological progression in patients with risk factors for rapid progression (i.e. high urinary C-terminal crosslinking telopeptide, high urinary pyridinoline/deoxypyridinoline ratio, low body mass index, and presence of joint space narrowing at baseline) than those without the risk factors. Furthermore, early decreases in serum type IIA procollagen amino terminal propeptide, CRP, and/or matrix metalloproteinase 3 (MMP-3) within 12 weeks can predict for the preventive effects of TCZ on one year progression of joint destruction in RA.

Although long-term treatment with TCZ is well tolerated, it goes without saying that it is beneficial not only for the patients but also for medical economy. To test the possibility of drug free remission introduced by TCZ, Drug free REmission after cessation of Actemra Monotherapy (DREAM) study was conducted. A total of 187 patients, who had received TCZ in the previous clinical trials (the mean treatment duration was 4.3 years), were enrolled, and discontinued TCZ. Remission, defined as DAS28 less than 2.6, was maintained in 10% of the patients without any drug over 52 weeks. Furthermore, low serum IL-6 (<35 pg/mL) and normalization of MMP-3 levels at cessation of TCZ were identified as independent predictive markers for the longer duration of drug free remission. In addition, retreatment with TCZ in the patients, who responded to initial TCZ monotherapy, and experienced loss of efficacy after cessation of TCZ, was well tolerated and showed excellent efficacy equivalent to that observed at the initial treatment with TCZ.

In the near future, tailor made therapy for individual patients will be developed on the basis of genome wide association study results, gene expression profile in peripheral blood cells and/or various biomarkers.

